# Predictors of Non-alcoholic Fatty Liver Disease in Obese and Overweight Egyptian Children: Single Center Study

**DOI:** 10.4103/1319-3767.74476

**Published:** 2011

**Authors:** Hanaa M. El-Karaksy, Nehal M. El-Koofy, Ghada M. Anwar, Fatma M. El-Mougy, Ahmed El-Hennawy, Mona E. Fahmy

**Affiliations:** Department of Pediatrics, Cairo University, Research Institute of Ophthalmology, Egypt; 1 Department of Chemical Pathology, Cairo University, Research Institute of Ophthalmology, Egypt; 2 Department of Pathology, Cairo University, Research Institute of Ophthalmology, Egypt

**Keywords:** Children, Egypt, NAFLD, NASH, obesity, steatosis

## Abstract

**Background/Aim:**

Pediatric non-alcoholic fatty liver disease (NAFLD) is a global problem which has been increasingly recognized with the dramatic rise in pediatric obesity. The aim of the present study was to identify the clinical, sonographic, and biochemical predictors for NAFLD in obese children.

**Materials and Methods:**

Seventy-six children (2-15 years) were included after an informed consent. All were subjected to full anthropometric assessment (including height, weight, body mass index, subscapular skin fold thickness, waist and hip circumference and calculation of waist: hip ratio), biochemical assessment of liver function tests, lipid profile and insulin resistance and sonographic assessment of hepatic echogenicity. Liver biopsy when indicated, was done in 33 patients.

**Results:**

Sixteen patients (21%) had elevated ALT and 6 (7.9%) had elevated AST. Significant dyslipidemia (low HDL-c, high total cholesterol, high LDL-c and triglycerides) and higher insulin resistance were found in obese patients (*P*<0.01). The main sonographic findings were hepatomegaly in 20 patients (26.3%) and echogenic liver in 41 patients (53.9%). Liver biopsy showed simple steatosis in eight cases (24.2%) and non-alcoholic steatohepatitis (NASH) in seven cases (21.2%). Anthropometric measurements, increased hepatic echogenicty by ultrasound, insulin resistance and lipid profile were good predictors of NAFLD in obese children if assessed together. However, LDL-c was the only sensitive predictor (independent variable) for NAFLD in both uni- and multivariate logistic regression analyses.

**Conclusion:**

Dyslipidemia per se is a strong predictor of NAFLD among obese Egyptian children.

Pediatric non-alcoholic fatty liver disease (NAFLD) is considered as a global problem which has increased in prevalence with the dramatic rise in obesity in children during the past three decades. Nevertheless, it cannot be excluded that this may partially represent increased recognition of this condition.[1]

Apart from obesity, several risk factors were reported to be associated with NAFLD; such as diabetes, insulin resistance (IR), and hypertriglyceridemia.[2]

NAFLD is an important cause of chronic liver disease in children. The spectrum of NAFLD ranges from simple steatosis, non-alcoholic steatohepatitis (NASH) with or without fibrosis to advanced fibrosis and cirrhosis when fat may no longer be present.[3]

Paralleling the increasing prevalence of obesity and type 2 diabetes in the pediatric population, NAFLD, especially its more severe histological form-NASH, is expected to become one of the most common causes of end-stage liver disease in both children and young adults.[4] Liver biopsy remains the most sensitive and specific means of providing important diagnostic and prognostic information.[5] Nevertheless, the cost of liver biopsy and the complications directly related to the procedure or to the sedation are reported to be higher (up to 18%) in infants than in adults.[6]

Recently, several studies investigated clinical and laboratory parameters which may help in predicting NAFLD. These predictors may be useful in guiding selection of patients for liver biopsy and in targeting therapy.

In adults, Singh *et al*[7] reported that female gender, body mass index (BMI), waist: hip (W/H) ratio, hypercholesterolemia and low density-lipoprotein (LDL) levels are independent predictors of disease severity in patients with NASH and may influence the decision to conduct biopsy.

Park and colleagues[8] reported that a high BMI with a low alanine aminotransferase (ALT) value tended to suggest the presence of severe fibrosis in NASH.

In a cohort of 69 children with NASH, Iacobellis *et al*[4] found that BMI helps identify children with NASH who might have fibrotic deposition in the liver.

The aim of the present study was to identify the clinical, sonographic, and biochemical predictors of NAFLD in obese children.

## MATERIALS AND METHODS

This cross-sectional study included 76 obese and overweight children referred to the Endocrine Unit at Cairo University Children’s Hospital. The study was approved by the Institutional Review Board. The patients were enrolled after an informed consent was obtained from the parent(s).

### Inclusion criteria

- Simple obesity and overweight
- Both sexes
- Age 2-15 years

### Exclusion criteria

- Patients with known disorders to cause fatty liver (e.g., HCV, diabetes, glycogen storage disease, and Wilson’s disease)
- Long term use of drugs known to cause steatosis (e.g. glucocorticoids, aspirin)
- Any case with syndromatic obesity

### Anthropmetric measurements

Patients’ anthropometric measurements including height (Ht), weight (Wt), and BMI were plotted on Egyptian growth curves.[9] Children were defined as overweight if their BMI was equal to or above 85th percentile and as obese if BMI was equal to or above 95th percentile.[9] Subscapular skins fold thickness (SSFT) was measured by skin fold caliper (Holtain LTD, UK) and was plotted on Egyptian growth curves. Waist circumference (WC) was measured at the level midway between the lowest rib margin and the iliac crest and was plotted on American percentiles for waist circumference.[10] Hip circumference was measured at the widest level over the greater trochanters in a standing position, by the same examiner; then calculation of W/H ratio was done. Abnormal W/H is considered if >0.86.

### Laboratory tests

All patients underwent the following laboratory tests (following not less than 12-h fasting period):

ACTH: using an auto-analyzer (DPC/immulite Gamma trade-USA) (normal range 2.0-8.0 pg/ml).

Cortisol: by radioimmunoassay (DPC-USA) (normal range less than 3.0 *μ*g %).

Both ACTH and cortisol were done after single dose dexamethazone suppression test (20 *μ*g/kg at the night before the test) to exclude Cushing syndrome.

Total cholesterol (normal range 100-200 mg/dl), high density lipoprotein-cholesterol (HDL-c) (normal range 30-70 mg/dl), low-density lipoprotein-cholesterol (LDL-c) (normal range less than 130 mg/dl), triglycerides (TG) (normal range 35-160 mg/dl), and fasting blood sugar (FBS) (normal range 65-100 mg/dl). Measurement was carried out using an auto-analyzer (Synchron-clinical system-CX5).

Fasting serum insulin and C-peptide measurement by an auto-analyzer (DDC/immulite).

Insulin resistance was calculated using the following equation:

The homeostasis model assessment method HOMA= [fasting insulin (*μ*U/ml) × fasting glucose (mmol/l)/22.5]

Liver functions: Total serum bilirubin (normal range: 0.2-1 mg/dl), direct serum bilirubin (normal range 0.1-0.3 mg/dl), alanine aminotransferase (ALT) (normal range 5-41U/l), aspartate aminotransferase (AST) (normal range 5-37 U/l), alkaline phosphatase AP (normal range 180-1200 U/l), and gamma glutamyl transpeptidase (GGT) (normal range 0-50 U/l). These tests were measured by the auto-analyzer (Abbott AXSYM system-UK).

Prothrombin time (PT) and concentration (PC) (normal range: 75-100%).

Serum ferritin (normal range: male 30-233 ng/ml, female 6-81 ng/ml) by an auto-analyzer (AxSYM Ferritin-Abbott laboratories, Abbott Park, IL, USA)

### Complete blood count

Children having raised transaminases underwent testing for hepatitis B and C viral markers (HBsAg, HBcAb and HCVAb). HBV markers were tested by enzyme-linked immunosorbent assay, IMX Surface/Core Assay, Germany. HCVAb was tested by enzyme-linked immunosorbant assay, DIA. PRO, Milano, Italy.

### Abdominal ultrasound examination

Was performed for all enrolled patients by a single sonographer following not less than eight hours fasting using an FFsonic UF-4100.

Liver echo pattern was graded as follows.[11]

Grade 1 (mild): A slight diffuse increase in fine echoes in the hepatic parenchyma with normal visualization of the diaphragm and intrahepatic vessel borders

Grade 2 (moderate): A moderate diffuse increase in fine echoes with slightly impaired visualization of the intrahepatic vessels and diaphragm

Grade 3 (marked): A marked increase in fine echoes with poor or no visualization of the intrahepatic vessel borders, diaphragm, and posterior portion of the right lobe of the liver

### Liver biopsy

Forty one cases had clinical hepatomegaly and/or elevated liver enzymes and/or increased liver echogenicity by ultrasonography and were candidates for liver biopsy. However, it was obtained in 33 patients only, with parental refusal in 8 cases. Histopathological examination of the specimens was carried out by a single pathologist.

The main histological features commonly described in NALFD/NASH, including steatosis, inflammation (portal and lobular), hepatocyte ballooning, and fibrosis, were scored according to the scoring system for NAFLD.[12]

### Controls

Twenty healthy control subjects, age and sex matched were included for insulin and C-peptide estimation after an informed consent.

### Statistical methods

Statistical Package for Social Science (SPSS) program version 9.0 was used. Data were summarized as mean and SD. The t-test was used for comparison of two independent variables (when data were found to be symmetrically distributed), while the non-parametric test (Mann Whitney) was used when data were not symmetrically distributed.

One way ANOVA was used for analysis of more than two independent variables followed by the *posthoc* test for detection of significance. *P*-value was considered significant if < 0.05.

## RESULTS

Out of the 76 patients included in the study, 37 were overweight (17 males and 20 females) and 39 were obese (21 males and 18 females). Their ages ranged between 2 and 15 years and the mean age was 7.7±3.5 years.

Among the study population, 74 (97.4 %) had WC >90^th^. Twenty-four (32%) had W/H ratio=1, while the remaining 52 (68%) cases had their W/H ratio = 0.9. The SSFT was ≥97^th^ in 58 (76 %), =95^th^ in 13 (17%) and = 90^th^ in 5 (7%). Five patients had clinical hepatomegaly.

Hormone and lipid profiles of 76 patients are summarized in Table 1.

Comparison between our patients and controls as regards insulin, C-peptide, and HOMA-IR showed statistically higher values in patients (*P*= 0.0001).

**Table 1:** Laboratory data of the studied groups

Variable	Overweight N=37 (mean ± SD)	Obese N=39 (mean ± SD)	*P* value	Total studied cases N=76 (mean ± SD)	Abnormal tests N (%)
Cortisol (μg/dl)	1.5 ± 0.5	1.4 ± 0.6	0.6	1.5 ± 0.5	0 (0)
ACTH (pg/ml)	5.3 ± 1	4.9 ± 1.1	0.1	5.1 ± 1.1	0 (0)
Fasting blood glucose (mg/dl)	88.7 ± 8.2	89.7 ± 8.4	0.6	89.2 ± 8.3	9 (11.8)
Total cholesterol (mg/dl)	170 ± 19.9	175.4 ± 22.4	0.3	172.8 ± 21.3	24 (31.6)
Triglyceride (mg/dl)	112.7 ± 38.7	134.4 ± 52.3	0.05^*^	124.1 ± 47.3	25 (32.9)
HDL-c (mg/dl)	47.4 ± 9.8	41.8 ± 9.2	0.01^*^	44.4 ± 9.8	10 (13.2)
LDL-c (mg/dl)	79.1 ± 19.7	87.8 ± 17.9	0.02^*^	83.7 ± 19.1	3 (3.9)
Insulin (µIU/ml)	13.4 ± 4.7	15.2 ± 6.9	0.3	14.3 ± 6.0	23 (30.3)
C-peptide (ng/ml)	1.4 ± 0.7	1.8 ± 0.8	0.03^*^	1.6 ± 0.8	21 (27.6)
HOMA-IR	3.1 ± 0.9	4.3 ± 0.8	0.0001^*^	3.2 ± 1.4	21 (27.6)
Total serum bilirubin (mg/dl)	0.5 ± 0.3	0.6 ± 0.2	0.2	0.5 ± 0.2	0 (0)
Direct serum bilirubin (mg/dl)	0.4 ± 0.2	0.4 ± 0.1	0.7	0.4 ± 0.2	0 (0)
AST (IU/l)	27.7 ± 15.2	30.5 ± 9	0.02^*^	29.2 ± 12.3	6 (7.9)
ALT (IU/l)					
Median	24	36.5	0.0001^*^	31.1 ± 23.9	16 (21)
Min-max	13-218	16-55			
AP (IU/l)	297.9 ± 73.9	341.8 ± 64.6	0.006^*^	321 ± 72.1	0 (0)
GGT (IU/l)	28.6 ± 17.2	30.7 ± 10.9	0.1	29.7 ± 14.2	0 (0)
Serum albumin (g/dl)	4.3 ± 0.2	4.3 ± 0.2	0.2	4.3 ± 0.2	0 (0)
PC(%)	85.6 ± 5	88 ± 7.2	0.2	86.9 ± 6.4	0 (0)
Serum ferritin(μg/l)	51.8 ± 21.1	61.8 ± 36.2	0.3	57.1 ± 30.3	0 (0)
Biopsy proven NAFLD (N=33)	0 (0)	15 (38.5)	<0.0001	33/76	15 (45.5)

Figures in parenthesis are in percentage

Sixteen patients (21%) had elevated ALT and 6 patients (7.9%) had elevated AST, otherwise, GGT, AP, total serum protein, serum albumin, total and direct serum bilirubin levels, ferritin and prothrombin concentration were within normal limits Table 1.

Comparison of data of overweight (n=37) and obese (n=39) patients are shown in Table 1. Lipid profile, insulin resistance, and transaminases showed statistically significant difference between both groups.

The abnormal sonographic findings were hepatomegaly in 20 patients (26.3%) and echogenic liver in 41 patients (53.9%); 17 patients (22.4%) showed grade I echogenicity, 14 (18.3%) grade II echogenicity, and 10 (13.2%) grade III echogenicity.

The lipid profile and hormonal values were also compared between these three groups and data are shown in Table 2.

**Table 2:** Comparison of data between three patient groups with grades I, II, and III hepatic echogenicity

Variable	Grade I (Mean ± SD) (n= 17)	Grade II (Mean ± SD) (n= 14)	Grade I (Mean ± SD) (n= 10)	*P* value
BMI	29.2 ± 1.7a	33 ± 4^b^	37.3 ± 6.6^c^	0.0001*
W/H ratio	0.9 ± 0.03^a^	1 ± 0.04^b^	1 ± 0.02^b^	0.0001*
Cortisol (μg/dl)	1.4 ± 0.5	1.4 ± 0.6	1.6 ± 0.6	0.7
ACTH (pg/ml)	4.9 ± 1.4	5 ± 1.3	4.9 ± 0.9	0.9
Fasting blood glucose (mg/dl)	89.8 ± 8	89.3 ± 10.6	95.2 ± 6.4	0.2
Total cholesterol (mg/dl)	167.8 ± 22.6^a^	179.0 ± 20.6^a^	194.9 ± 7.7^b^	0.005*
Triglyceride (mg/dl)	118.9 ± 38.0^a^	135.1 ± 54.4^a^	190.9 ± 30.5^b^	0.001*
HDL-c (mg/dl)	46.1 ± 9.6^a^	40.1 ± 7.0^b^	34.1 ± 5.3^b^	0.002*
LDL-c (mg/dl)	80.9 ± 24.4^a^	91.3 ± 16.2^b^	102.2 ± 16.1^c^	0.03*
Insulin (μIU/ml)	12.5 ± 5.4^a^	15.8 ± 8.7^ab^	19.6 ± 5.4^b^	0.04*
C-peptide (ng/ml)	1.4 ± 0.6^a^	1.8 ± 0.9^a^	2.6 ± 0.5^b^	0.001*
HOMA-IR	2.83 ± 1.33^a^	3.44±1.70^ab^	4.11 ± 1.16^b^	<0.05*
Total serum bilirubin (mg/dl)	0.6 ± 0.3	0.6 ± 0.2	0.5 ± 0.2	0.5
Direct serum bilirubin (mg/dl)	0.5 ± 0.2	0.4 ± 0.1	0.3 ± 0.1	0.2
AST (IU/l)	32.6 ± 21.1	27.1 ± 6.2	37.3 ± 9.1	0.3
ALT (IU/l)	36.7 ± 8.4	33.9 ± 6.7	43.5 ± 9	0.8
AP (IU/l)	315.9 ± 55.6^a^	331.6 ± 56.2^a^	398.1 ± 57.1^b^	0.002*
GGT (IU/l)	32.5 ± 23.7	31.2 ± 8.1	38.6 ± 12.2	0.6
Serum albumin (gm/dl)	4.3 ± 0.2	4.4 ± 0.3	4.3 ± 0.2	0.3
PC (%)	87.1 ± 6.1	87.1 ± 8	91 ± 9.1	0.4
Serum ferritin (μg/l)	57.2 ± 1.9	65.1 ± 54.8	57.2 ± 19.6	0.8
Liver histology	N (%)	N (%)	N (%)	
Normal (n=18)	12 (66.7)	6 (33.3)	0 (0)	0.01*
Simple steatosis(n=8)	0 (0)	5 (62.5)	3 (37.5)	
NASH (n=7)	0 (0)	0 (0)	7 (100)	

*P* value is significant if <0.05^*^

Different symbols indicate signifi cant difference. Figures in parenthesis are in percentage

A percutaneous liver biopsy was obtained in 33 patients: 18 cases (54.6%) had normal liver histology, 8 (24.2%) had simple steatosis, and 7 (21.2%) had NASH. The highest BMI values were among those with NASH. The same was true as regards the lipid profile and liver function tests Table 3.

**Figure 1:**
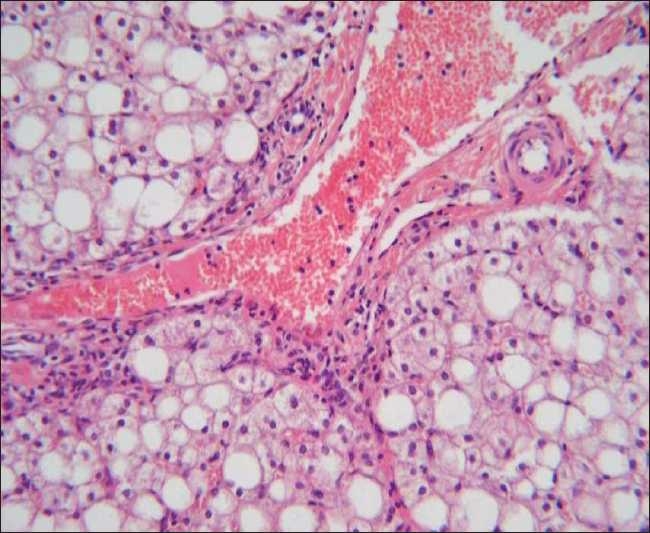
Liver biopsy specimen stained with hematoxylin and eosin (original magnification ×200) showing moderate macrovesicular fatty changes, mild necroinflammatory activity and grade I fibrosis

Patients with NASH were histologically graded. Four patients had moderate macrovesicular fatty changes and grade 1 (mild) necroinflammatory activity. Three patients had moderate macrovesicular fatty changes, grade 1 (mild) necroinflammatory activity, and stage 1 fibrosis Figure 1.

**Table 3:** Comparison of data of patients with normal liver histology, simple steatosis, and NASH

Variable	Normal histology (Mean ± SD) (n= 18)	Simple steatosis(Mean ± SD) (n= 8)	NASH (Mean ± SD) (n= 7)	*P* value
BMI	30.0 ± 2.2^a^	36.6 ± 4.3^b^	38.4 ± 6.4^b^	0.0001*
SSFT (cm)	25.1 ± 2.6^a^	34.3 ± 2.0^b^	31.6 ± 3.7^b^	0.0001*
W/H ratio	0.9 ± 0.03^a^	1.0 ± 0.08^b^	1.0 ± 0.2^b^	0.0001*
Total cholesterol (mg/dl)	170.2 ± 21.4^a^	191.4 ± 11.4^b^	194.1 ± 8.5^b^	0.004*
HDL-c (mg/dl)	44.1 ± 7.6^a^	36.9 ± 8.3^b^	33.6 ± 6.1^b^	0.007*
LDL-c (mg/dl)	78.6 ± 14.4^a^	100.0 ± 14.5^b^	104.9 ± 16.6^b^	0.0001*
Triglyceride (mg/dl)	124.9 ± 41.6^a^	170.8 ± 31.6^b^	195.9 ± 36^b^	0.001*
Insulin (μIU/ml)	12.4 ± 6.0^a^	20.2 ± 8.5^b^	19.6 ± 6.4^b^	0.02*
C-peptide (ng/ml)	1.4 ± 0.6^a^	2.3 ± 0.8^b^	2.7 ± 0.5^b^	0.0001*
HOMA-IR	3.1 ± 0.9^a^	4.3 ± 0.8^b^	4.1 ± 0.8^b^	0.0001*
Total bilirubin (mg/dl)	0.4 ± 0.1^a^	0.6 ± 0.1^a^	0.7 ± 0.2^b^	0.01*
Direct bilirubin (mg/dl)	0.3 ± 0.1^a^	0.4 ± 0.1^a^	0.4.1 ± 0.1^b^	0.02*
AST (U/l)	27.3 ± 9.2^a^	30.0 ± 8.5^ab^	37.6 ± 8.9^b^	0.04*
ALT (U/l)	28.6 ± 7.4^a^	40.8 ± 6.0^b^	44.6 ± 6.1^b^	0.0001*
AP (U/l)	319.4 ± 56.8^a^	370.5 ± 39.8^b^	404.1 ± 68.1^b^	0.004*
GGT	26.3 ± 8.0^a^	33.3 ± 10.5^a^	43.4 ± 8.3^b^	0.002*
Serum albumin (g/dl)	4.4 ± 0.2	4.4 ± 0.3	4.2 ± 0.3	0.3
PC (%) 85.6 ± 5.4	91.9 ± 8.4	90.7 ± 9.3	0.08
Serum ferritin (μg/l)	55.2 ± 21.9	88.4 ± 64	53.78 ± 19	0.5

*P* value is significant if < 0.05*

Different symbols indicate signifi cant difference. Figures in parenthesis are in percentage

By binary logistic regression analysis, W/H ratio, BMI and SSFT, liver echogenicity, insulin, C-peptide, and cholesterol were all good predictors for NAFLD in univariate analysis. *P* value was <0.01 for each. However, in multivariate analysis, they could no longer predict NAFLD (*P*>0.05 for each).

Only LDL was a good predictor for NAFLD in both univariate and multivariate analyses, *P* value <0.01.

## DISCUSSION

To the best of our knowledge, this is the first Egyptian study determining the prevalence and predictors of NAFLD in obese children and adolescents. Our study shows that the estimated prevalence of NAFLD is 38.5% in obese Egyptian children and adolescents.

It is still less than that reported by other researchers. In a series of Canadian children from multiethnic origin with body weight ranging from 114 to 192% of ideal weight-for-height, 71% had features of fibrosis.[13] Ong *et al*[14] found that 93% of the 212 morbidly obese patients had NAFLD. Xanthokos *et al*[15] studied the histological spectrum of 41 obese American adolescents and found that 83% had NAFLD; 24% had steatosis alone, 7% isolated fibrosis with steatosis, 32% nonspecific inflammation and steatosis, and 20% NASH.

The higher prevalence rates in other studies can be explained by the fact that they included obese children; however, our study included both overweight and obese children. Also in our study, HBV and HCV antibodies positive patients were excluded from the study.

In the present work, many risk factors have been studied to determine their relation and predictive values to NAFLD.

First, the degree of obesity, represented by BMI, SSFT, and W/H ratio was probed. BMI, SSFT and W/H ratio were good predictors for NAFLD in univariate logistic regression analysis. This can be explained by the fact that obese patients have significantly higher dyslipidemia and insulin resistance; both can contribute to hepatic steatosis and these results are in agreement with Finucane *et al*.[16] A multivariate analysis showed that BMI was the only independent predictor of fibrosis in children with NASH.[4]

Second, insulin resistance was assessed by measuring fasting serum insulin level, C-peptide, and calculating HOMA-IR. In the current study, they were significantly higher in obese patients with NAFLD, and they were good predictors for NAFLD in univariate logistic regression analysis. This comes in agreement with other studies.[16,17] Almost all patients with NASH are insulin resistant.[18] Insulin resistance plays a key role in the development of NASH.[19]

Third, the present study revealed a significant dyslipidemia (lower HDL-c, higher total cholesterol, LDL-c and triglycerides) in obese patients with grade II-III liver echogenicity and in those with NAFLD. Total cholesterol in univariate logistic regression analysis was a good predictor of NAFLD; however, LDL-c was a good predictor in both uni- and multivariate analysis. Association of dyslipidemia with NAFLD in this study comes in agreement with other studies.[20,21]

Fourth, liver enzymes (ALT, AST, GGT, and ALP) were significantly higher in obese patients with NAFLD. Although ALT carries the highest significant difference between patients with and without hepatic steatosis, no significant difference has been found between ALT levels in patients with simple steatosis and those with NASH. So measurement of liver enzymes alone is not sufficient to diagnose NASH.[15,17]

Fifth, a significant correlation has been found between the degree of liver echogenicity on ultrasonography and hepatic steatosis. Liver echogenicity is a good predictor of NAFLD in univariate logistic regression analysis. Seventy percent of our patients who showed grade III liver echogenicity were diagnosed as NASH by liver biopsy, and the remaining 30% were simple steatosis. Although ultrasonography cannot distinguish fat from fibrous tissue,[22] along with other clinical and biochemical parameters it will be of help in selecting patients who are in need of liver biopsy.

Hepatomegaly was detected in 20 cases (26%) by ultrasonography; however, clinical hepatomegaly was palpable in only five cases (6.5%). This is because in most obese patients it is difficult to palpate abdominal organs due to excessive abdominal fat and pendulous abdomen.[23]

Because most of the studied parameters, each on its own, cannot predict NAFLD, scores have been developed recently for NAFLD prediction.[24]

From our results, we can conclude that anthropometric measurements including BMI, SSFT, and W/H ratio together with ultrasonographic assessment of hepatic ecchogenicty and laboratory variables including (insulin resistance and dyslipidemia) are good predictors of NASH among obese children. These predictors are sensitive if assessed together. Only LDL-c is a sensitive predictor for NASH in both uni- and multivariate logistic regression analyses which makes dyslipidemia per se a strong predictor of NASH among obese children.
